# Supplementary material for: Plasma metabolomic profiles associated with mortality and longevity in a prospective analysis of 13,512 individuals
Source: Nat Commun. 2023 Sep 16;14:5744. doi: 10.1038/s41467-023-41515-z (PMC10505179; doi:10.1038/s41467-023-41515-z)
Supplement: Supplementary file 1 — Supplementary Information [file 41467_2023_41515_MOESM1_ESM.pdf]

Supplementary Information for

Plasma metabolomic profiles associated with mortality and longevity in a prospective analysis of 13,512 individuals

Fenglei Wang[1], Anne-Julie Tessier[1], Liming Liang[2,3], Clemens Wittenbecher[1,4], Danielle E. Haslam[1,5], Gonzalo Fernández-Duval[6], A. Heather Eliassen[1,2,5], Kathryn M. Rexrode[7,8], Deirdre K. Tobias[1,7], Jun Li[1], Oana Zeleznik[5], Francine Grodstein[9], Miguel A. Martínez-González[1,10,11], Jordi Salas-Salvadó[10,12,13], Clary Clish[14], Kyu Ha Lee[1,2,3], Qi Sun[1,2,5], Meir J. Stampfer[1,2,5], Frank B. Hu[1,2,5,*], Marta Guasch-Ferré[1,15,16,*]

This PDF file includes:

Supplementary Figures

Supplementary Figure 1. Subgroup analyses and sensitivity analyses for the metabolome-wide associations of all-cause mortality
Supplementary Figure 2. Associations of metabolites or multi-metabolite profile score with cardiovascular mortality with and without adjusting for incident CVD
Supplementary Figure 3. Associations of metabolite groups and modules with cardiovascular and cancer mortality
Supplementary Figure 4. Topological overlap map of metabolites
Supplementary Figure 5. Metabolome-wide associations for longevity by sex and by different definitions for males
Supplementary Figure 6. External replication for the associations with all-cause mortality in PREDIMED

Supplementary Tables

Supplementary Table 1. Number of participants in the 13 prior sub-studies on metabolomics in NHS/NHSII/HPFS
Supplementary Table 2. Baseline characteristics of participants by death status in NHS/NHSII/HPFS and PREDIMED
Supplementary Table 3. Baseline characteristics of participants included in the longevity analysis

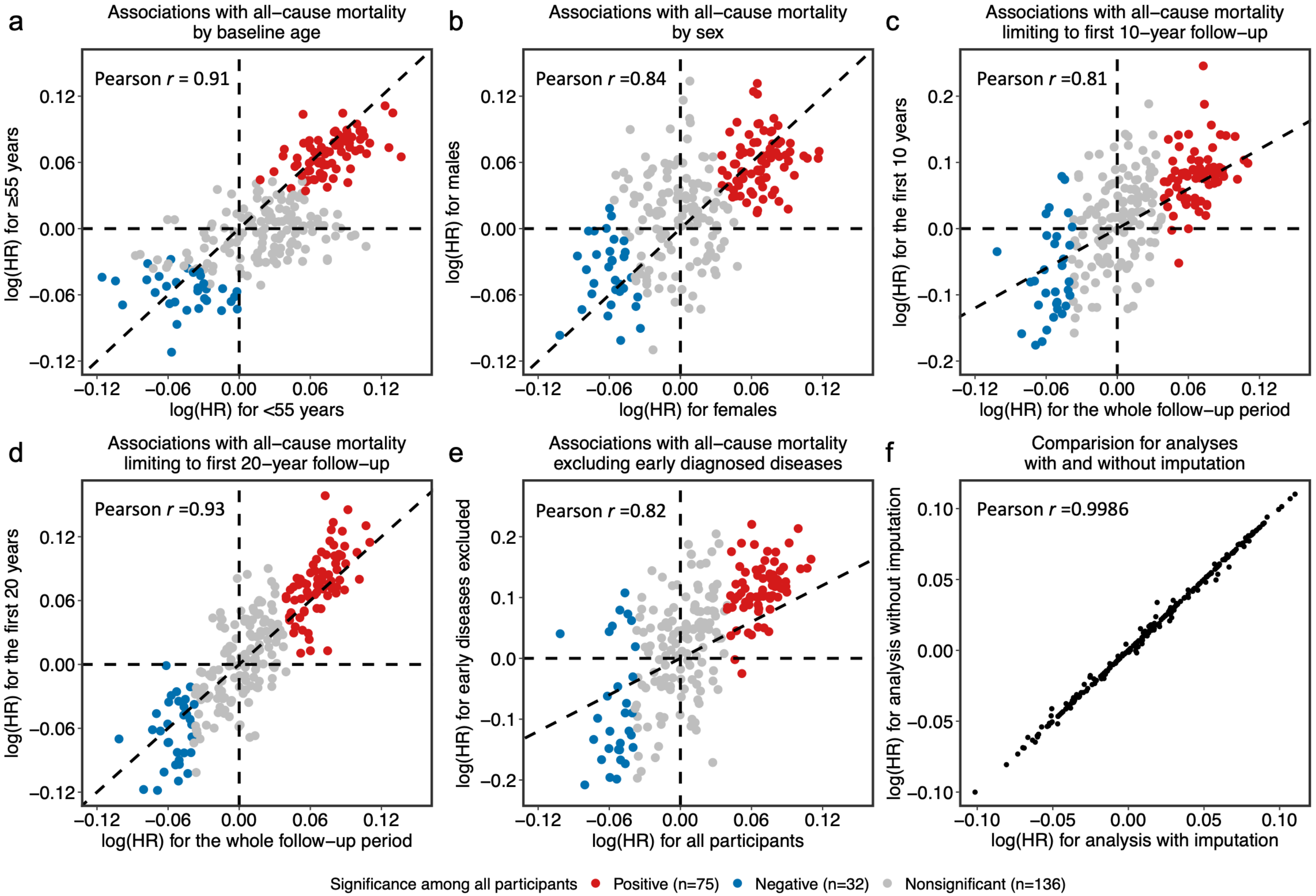

Supplementary Figure 1. Subgroup analyses and sensitivity analyses for the metabolome-wide associations of all-cause mortality. a). Subgroup analysis by baseline. **b).** Subgroup analysis by sex. **c).** Sensitivity analysis limiting to the first 10 years' follow-up. **d).** Sensitivity analysis limiting to the first 20 years' follow-up. **e).** Sensitivity analysis after excluding incident cardiovascular disease and cancer diagnosed within first 4

years after blood collection. **f).** Sensitivity analysis without imputing missing metabolite data (complete case analysis). All results were from the multivariable model stratified by study cohorts, original sub-studies, and the case/control status in the original sub-study, and adjusted for age, fasting status, body mass index, race, multivitamin use, smoking status, physical activity, diabetes, hypertension, antihypertensive medication use, hypercholesterolemia, lipid-lowering medication use, total energy intake, alcohol intake, and Alternate Healthy Eating Index. Source data are provided as a Source Data file.

Metabolite	Without adjusting for incident CVD<br>HR (95% CI)	Adjusting for incident CVD<br>HR (95% CI)	Proportion attributable to incident CVD, %	P
**NHS/NHSII/HPFS**				
4-Acetamidobutanoic acid	1.12 (1.05, 1.20)	1.05 (0.98, 1.12)	59.1	<0.001
C36:3 PE*	1.10 (1.03, 1.18)	1.05 (0.98, 1.12)	52.4	0.007
C46:2 TAG*	1.10 (1.03, 1.19)	1.05 (0.98, 1.14)	47.7	0.01
C48:3 TAG*	1.10 (1.03, 1.18)	1.05 (0.98, 1.13)	45.5	0.01
C32:1 DAG*	1.13 (1.05, 1.22)	1.07 (0.99, 1.16)	41.0	0.01
C34:2 DAG*	1.11 (1.03, 1.19)	1.07 (0.99, 1.15)	35.8	0.03
C48:1 TAG*	1.11 (1.03, 1.20)	1.07 (0.99, 1.16)	34.2	0.04
C46:1 TAG*	1.11 (1.03, 1.20)	1.07 (1.00, 1.16)	32.7	0.04
C36:2 PE*	1.14 (1.07, 1.22)	1.10 (1.03, 1.17)	29.9	0.02
C38:5 PE*	1.15 (1.08, 1.22)	1.10 (1.04, 1.18)	28.0	0.04
C34:2 PE*	1.14 (1.07, 1.22)	1.10 (1.03, 1.17)	28.0	0.046
L-Serine	0.83 (0.76, 0.91)	0.87 (0.80, 0.95)	27.9	0.02
C34:1 DAG*	1.15 (1.07, 1.24)	1.11 (1.03, 1.19)	26.1	0.04
C16:0 Ceramide (d18:1)	1.15 (1.08, 1.23)	1.11 (1.04, 1.18)	25.4	0.04
Multi-metabolite profile score	1.32 (1.21, 1.44)	1.21 (1.10, 1.34)	30.5	<0.001
**PREDIMED**				
C16:0 Ceramide (d18:1)	1.39 (1.11, 1.74)	1.04 (0.78, 1.38)	89.4	0.001
4-Acetamidobutanoic acid	1.28 (1.01, 1.63)	1.09 (0.83, 1.43)	66.5	0.10
Multi-metabolite profile score	1.64 (1.26, 2.13)	1.35 (0.97, 1.89)	38.6	0.08

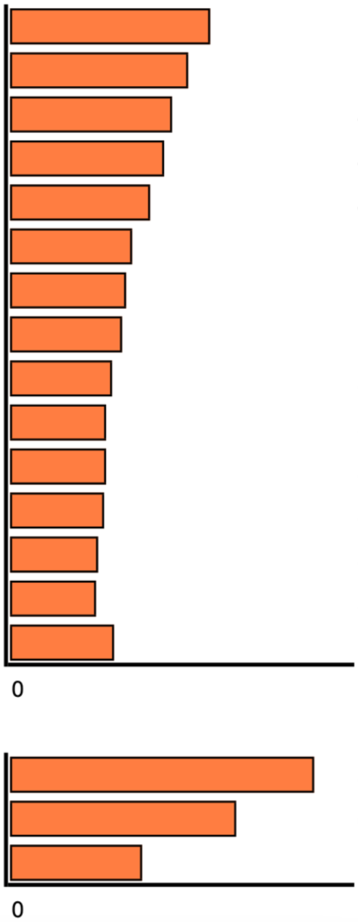

Supplementary Figure 2. Associations of metabolites or multi-metabolite profile score with cardiovascular mortality with and without adjusting for incident CVD. Results in NHS/NHSII/HPFS were stratified by study cohorts, original sub-studies, and the case/control status in the original sub-study, and adjusted for age, fasting status, body mass index, race, multivitamin use, smoking status, physical activity, diabetes, hypertension, antihypertensive medication use, hypercholesterolemia, lipid-lowering medication use, total energy intake, alcohol intake, and Alternate Healthy Eating Index. Results in PREDIMED were stratified by recruitment center and intervention group, and adjusted for age, sex, body mass index, smoking status, physical activity, diabetes, hypertension, antihypertensive medication use, hypercholesterolemia, lipid-lowering medication use, total energy intake, alcohol intake, and Mediterranean Diet Adherence Screener. Proportion attributable to incident CVD was calculated from the mediation analysis, taking the metabolite or multi-metabolite profile score as the mediator. P value was obtained from the mediation analysis. For NHS/NHSII/HPFS, only metabolites with P <0.05 and attributable proportion>10% were shown. Full results were in Supplementary Table 5. For PREDIMED, the end of follow-up was set in 2010 as incident CVD information was not available after that. Given the limited number of CVD deaths (n=67), only associations of 4-acetamidobutanoic acid and C16:0 ceramide (d18:1) were examined. We reported unadjusted P values based on two-sided statistical tests. Metabolites with * indicate representative names. Source data are provided as a Source Data file. Abbreviations: CVD, cardiovascular disease.

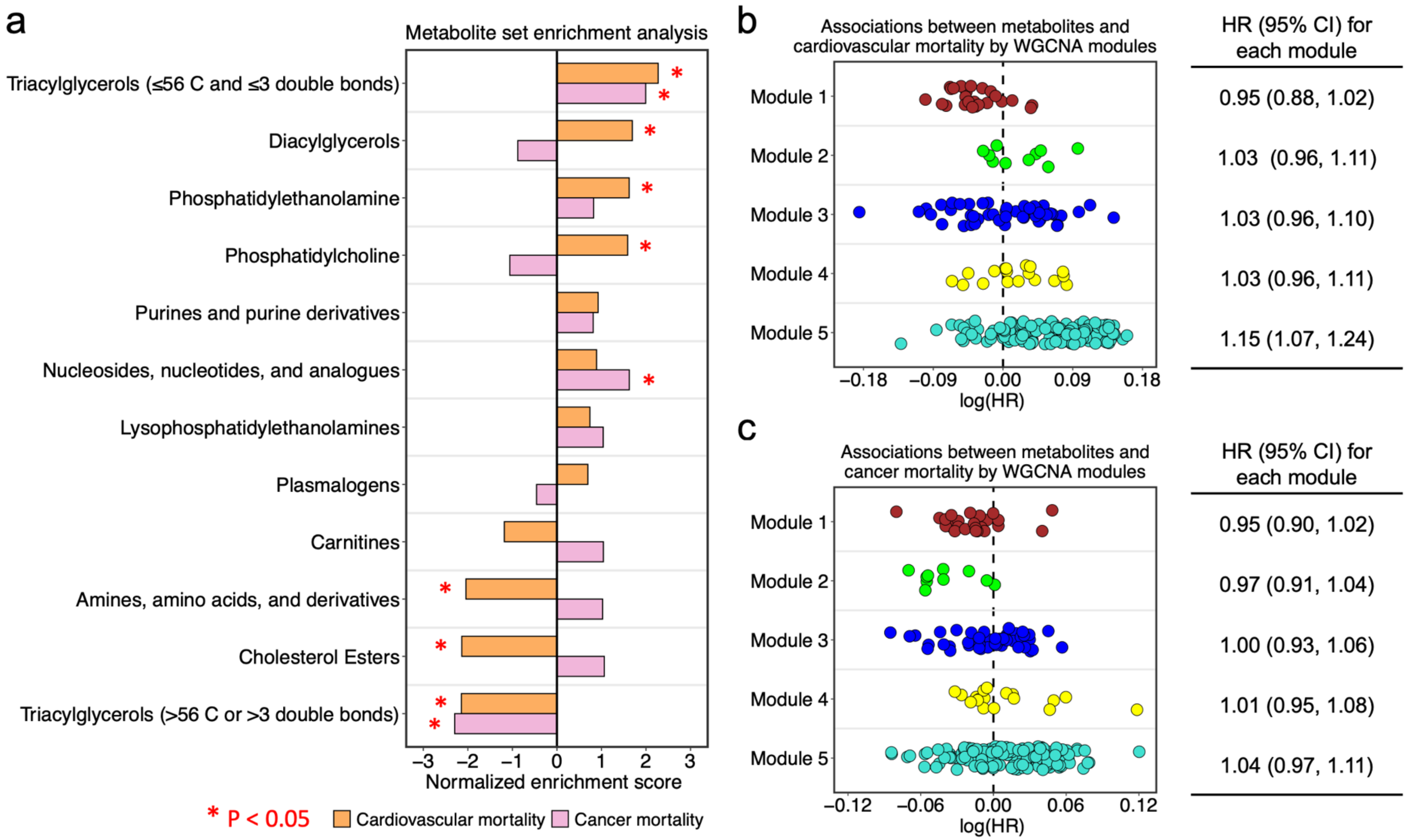

Supplementary Figure 3. Associations of metabolite groups and modules with cardiovascular and cancer mortality. a). Association between knowledge-based metabolite groups and cardiovascular and cancer mortality. Metabolite Set Enrichment Analysis was used to estimate enrichment scores based on estimates from multivariable Cox models. **b).** Metabolome-wide association for cardiovascular mortality by WGCNA derived modules, and association for each module, estimated using multivariable Cox models. **c).** Metabolome-wide association for cancer mortality by WGCNA derived modules, and association of each module, estimated using multivariable logistic regression models. All results were from the multivariable model stratified by study cohorts, original sub-studies, and the case/control status in the original sub-study, and adjusted for age, fasting status, body mass index, race, multivitamin use, smoking status, physical activity, diabetes, hypertension, antihypertensive medication use, hypercholesterolemia, lipid-lowering medication use, total energy intake, alcohol intake, and Alternate Healthy Eating Index. Source data are provided as a Source Data file. Abbreviation: WGCNA, weighted gene co-expression network analysis

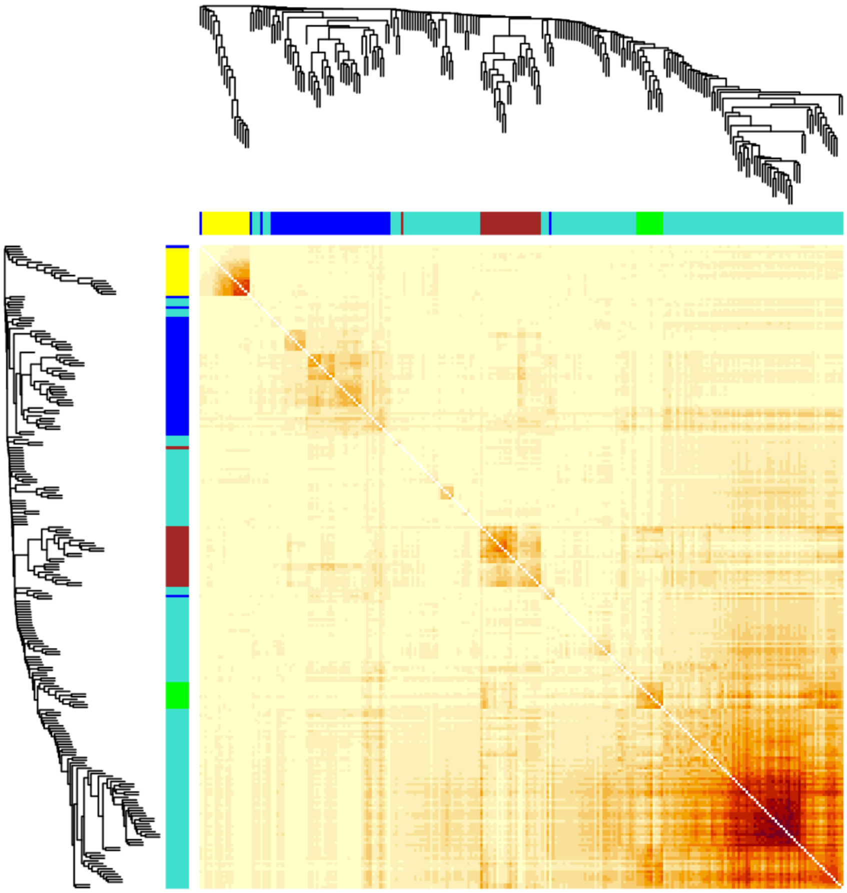

Supplementary Figure 4. Topological overlap map of metabolites. Metabolites in the rows and columns are sorted by the clustering tree. Light yellow shades represent low topological overlap (low similarity). Darker red shades represent higher overlap and similarity. Metabolite modules correspond to the squares along the diagonal.

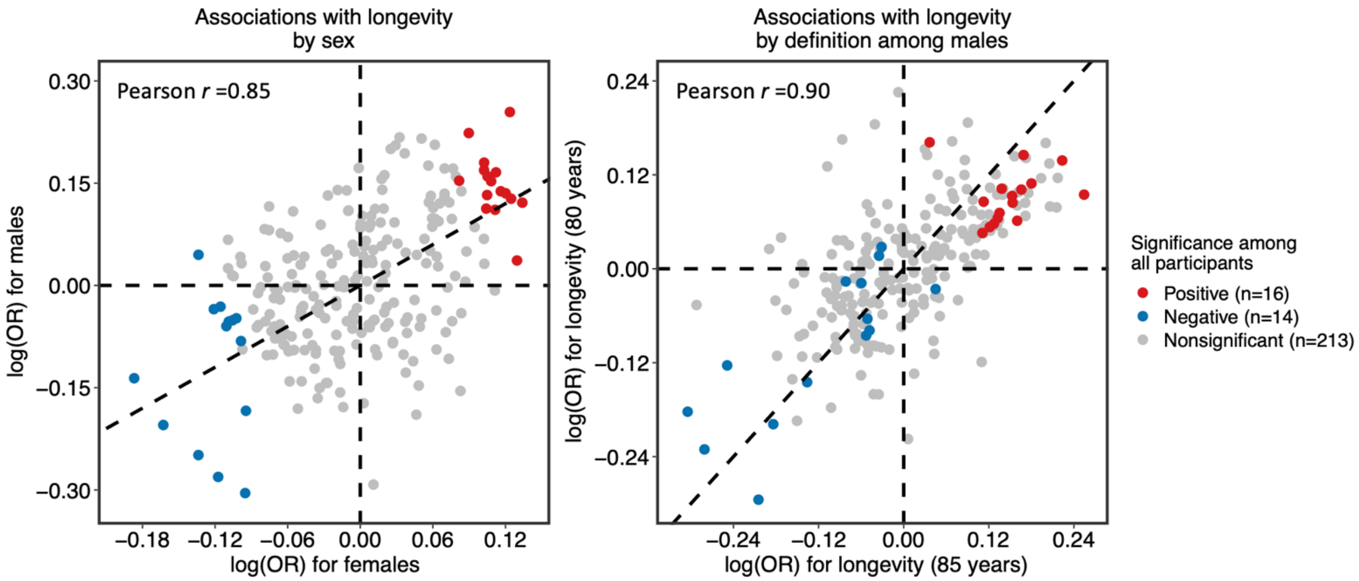

Supplementary Figure 5. Metabolome-wide associations for longevity by sex and by different definitions for males. All results were from the multivariable model adjusted for study cohorts, original sub-studies, case/control status in the original sub-study, age, fasting status, body mass index, race, multivitamin use, smoking status, physical activity, diabetes, hypertension, antihypertensive medication use, hypercholesterolemia, lipid-lowering medication use, total energy intake, alcohol intake, and AHEI. Source data are provided as a Source Data file.

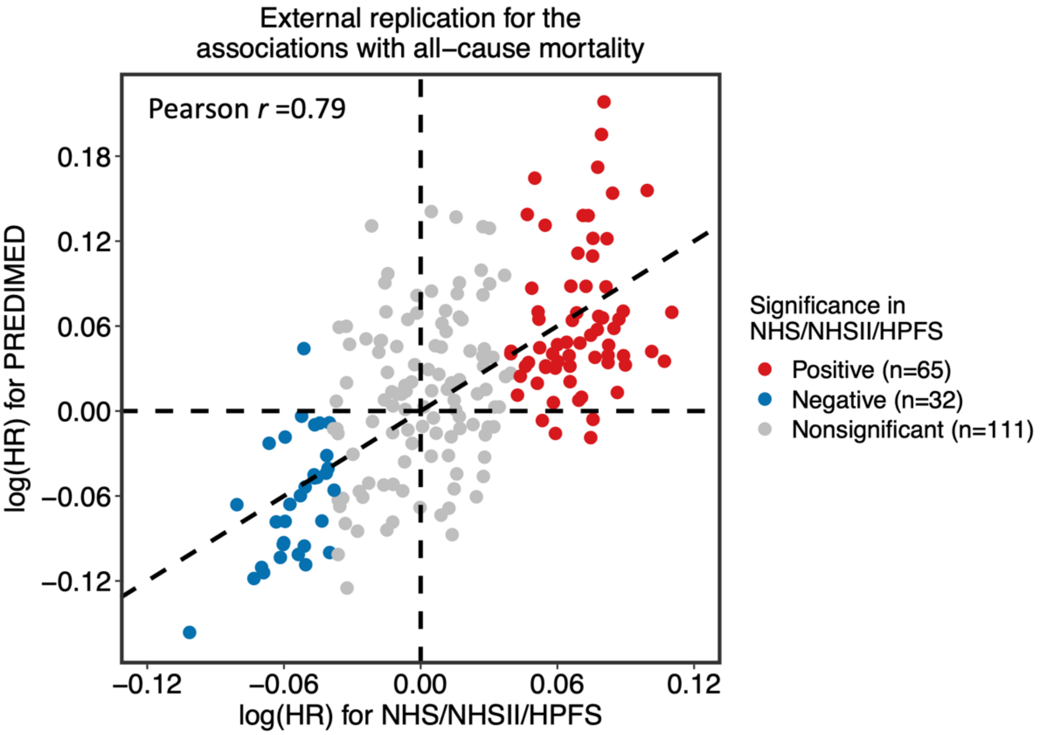

Supplementary Figure 6. External replication for the associations with all-cause mortality in PREDIMED. Results in PREDIMED were stratified by recruitment center and intervention group, and adjusted for age, sex, body mass index, smoking status, physical activity, diabetes, hypertension, antihypertensive medication use, hypercholesterolemia, lipid-lowering medication use, total energy intake, alcohol intake, and Mediterranean Diet Adherence Screener. Results in NHS/NHSII/HPFS were stratified by study cohorts, original sub-studies, and the case/control status in the original sub-study, and adjusted for age, fasting status, body mass index, race, multivitamin use, smoking status, physical activity, diabetes, hypertension, antihypertensive medication use, hypercholesterolemia, lipid-lowering medication use, total energy intake, alcohol intake, and Alternate Healthy Eating Index. Source data are provided as a Source Data file.

Supplementary Table 1. Number of participants in the 13 prior sub-studies on metabolomics in NHS/NHSII/HPFS

	NHS	NHSII	HPFS
Breast cancer	1668	2080	/
Colorectal cancer	691	/	408
Glaucoma (exfoliative)	326	/	/
Glaucoma (primary open-angle)	687	150	225
Inflammatory bowel disease	204	/	/
Mind body study	/	215	/
Ovarian cancer	465	/	/
Parkinson's disease	435	/	466
Prostate cancer	/	/	521
Racial difference	170	/	/
Rheumatoid arthritis	443	239	/
Stroke	459	112	/
Type 2 diabetes	1335	335	/
**Total**	6883	3131	1620

Supplementary Table 2. Baseline characteristics of participants by death status in NHS/NHSII/HPFS and PREDIMED

	**NHS/NHSII/HPFS**		**PREDIMED**	
	**Participants died during follow-up** (n=4288)	**Participants did not die during follow-up** (n=7346)	**Participants died during follow-up** (n=525)	**Participants did not die during follow-up** (n=1353)
Age, year	61.1 (7.3)	50.3 (7.2)	70.4 (5.9)	65.9 (5.6)
Female/male, %/%	77.6/22.4	91.0/9.0	47.0/53.0	61.5/38.5
Ethnicity (white), %	96.3	96.3	100	100
Fasting at blood collection, %	71.8	72.6	100	100
Body mass index, kg/m2	25.9 (4.7)	25.6 (5.0)	30.0 (3.7)	29.9 (3.5)
Physical activity (MET-hours/week)	19.4 (23.5)	18.2 (24.2)	4.2 (4.2)	4.1 (3.9)
Smoking status, %				
Never	44.0	57.0	52.8	61.9
Past	42.0	34.5	29.5	23.0
Current	14.0	8.4	17.7	15.1
Multivitamin use, %	63.8	68.5	/	/
Metabolic disease, %				
Diabetes	5.1	1.4	40.8	21.3
Hypertension	33.0	16.3	88.8	86.8
Hypercholesterolemia	33.9	24.6	61.9	79.7
Total energy intake, kcal/day	1823 (542)	1829 (544)	2345 (619)	2306 (595)
Alcohol intake, g/day	7.2 (12.2)	5.1 (9.1)	10.9 (18.1)	9.1 (14.5)
Alternate Healthy Eating Index (not including alcohol)	47.8 (10.3)	46.4 (10.0)	/	/

Values are means (SDs) for continuous variables and percentages for categorical variables.

Supplementary Table 3. Baseline characteristics of participants included in the longevity analysis

	NHS/NHSII/HPFS		PREDIMED	
	**Participants achieved longevity** (n=3067)	**Participants did not achieve longevity** (n=2230)	**Participants achieved longevity** (n=458)	**Participants did not achieve longevity** (n=407)
Age, year	63.7 (4.8)	58.7 (6.7)	73.8 (3.0)	68.9 (5.7)
Female/male, %/%	82.4/17.6	78.3/21.7	69.7/30.3	42.0/58.0
Ethnicity (white), %	96.3	96.0	100	100
Fasting at blood collection, %	74.5	70.6	100	100
Body mass index, kg/m2	25.3 (3.9)	26.2 (5.1)	29.9 (3.5)	30.1 (3.7)
Physical activity (MET-hours/week)	20.6 (26.7)	17.5 (19.9)	3.9 (3.5)	4.2 (4.3)
Smoking status, %				
Never	48.6	40.3	74.5	46.9
Past	43.6	41.3	18.1	32.7
Current	7.8	18.4	7.4	20.4
Multivitamin use, %	63.3	63.0	/	/
Metabolic disease, %				
Diabetes	2.8	6.0	26.9	41.0
Hypertension	30.4	33.7	90.0	88.7
Hypercholesterolemia	35.0	31.7	75.8	69.5
Total energy intake, kcal/day	1804 (529)	1825 (546)	2221 (532)	2380 (634)
Alcohol intake, g/day	6.8 (11.1)	7.4 (12.7)	7.7 (14.4)	12.1 (19.1)
Alternate Healthy Eating Index (not including alcohol)	49.3 (10.3)	46.8 (10.1)	/	/

Values are means (SDs) for continuous variables and percentages for categorical variables.
