# Supplementary material for: Plasma metabolomic profiles associated with mortality and longevity in a prospective analysis of 13,512 individuals
Source: Nat Commun. 2023 Sep 16;14:5744. doi: 10.1038/s41467-023-41515-z (PMC10505179; doi:10.1038/s41467-023-41515-z)
Supplement: Supplementary file 3 — Description of Additional Supplementary Files [file 41467_2023_41515_MOESM3_ESM.pdf]

File name: Supplementary Data 1
Description: Associations of metabolites with all-cause, cardiovascular, and cancer mortality in the fully adjusted model in NHS/NHSII/HPFS

File name: Supplementary Data 2
Description: Associations of metabolites or multi-metabolite profile score with all-cause, cardiovascular, and cancer mortality adjusting for incident CVD in NHS/NHSII/HPFS

File name: Supplementary Data 3
Description: Associations of metabolites or multi-metabolite profile score with all-cause, cardiovascular, and cancer mortality adjusting for incident cancer in NHS/NHSII/HPFS

File name: Supplementary Data 4
Description: Categories and module assignment of metabolites

File name: Supplementary Data 5
Description: Metabolites selected in the elastic net regression and their coefficients for all-cause mortality

File name: Supplementary Data 6
Description: Associations of metabolites with longevity in the fully adjusted model in NHS/NHSII/HPFS

File name: Supplementary Data 7
Description: Associations of metabolites with mortality and longevity in the fully adjusted model in PREDIMED
