# Supplementary material for: Plasma metabolomic profiles associated with mortality and longevity in a prospective analysis of 13,512 individuals
Source: Nat Commun. 2023 Sep 16;14:5744. doi: 10.1038/s41467-023-41515-z (PMC10505179; doi:10.1038/s41467-023-41515-z)
Supplement: Supplementary file 5 — Reporting Summary [file 41467_2023_41515_MOESM5_ESM.pdf]

nature portfolio

Corresponding author(s): Frank B. Hu and Marta Guasch-Ferré

Last updated by author(s): Jun 20, 2023

Reporting Summary

Nature Portfolio wishes to improve the reproducibility of the work that we publish. This form provides structure for consistency and transparency in reporting. For further information on Nature Portfolio policies, see our Editorial Policies and the Editorial Policy Checklist.

Statistics

For all statistical analyses, confirm that the following items are present in the figure legend, table legend, main text, or Methods section.

n/a	Confirmed	
☐	☒	The exact sample size (n) for each experimental group/condition, given as a discrete number and unit of measurement
☐	☒	A statement on whether measurements were taken from distinct samples or whether the same sample was measured repeatedly
☐	☒	The statistical test(s) used AND whether they are one- or two-sided<br>*Only common tests should be described solely by name; describe more complex techniques in the Methods section.*
☐	☒	A description of all covariates tested
☐	☒	A description of any assumptions or corrections, such as tests of normality and adjustment for multiple comparisons
☐	☒	A full description of the statistical parameters including central tendency (e.g. means) or other basic estimates (e.g. regression coefficient) AND variation (e.g. standard deviation) or associated estimates of uncertainty (e.g. confidence intervals)
☐	☒	For null hypothesis testing, the test statistic (e.g. F, t, r) with confidence intervals, effect sizes, degrees of freedom and P value noted<br>*Give P values as exact values whenever suitable.*
☒	☐	For Bayesian analysis, information on the choice of priors and Markov chain Monte Carlo settings
☒	☐	For hierarchical and complex designs, identification of the appropriate level for tests and full reporting of outcomes
☐	☒	Estimates of effect sizes (e.g. Cohen's d, Pearson's r), indicating how they were calculated

Our web collection on statistics for biologists contains articles on many of the points above.

Software and code

Policy information about availability of computer code

Data collection	No software was used for data collection.
Data analysis	All analyses were performed in R version 4.1.0. The main R packages used were 'missRanger 2.1.5' for random forest imputation, 'survival 3.4-0' for Cox regression, 'fgsea 1.23.2' for MSEA, 'WGCNA 1.71' for metabolite module derivation, and 'glmnet 4.1-4' for elastic net regression. The analysis programs are publicly available at

For manuscripts utilizing custom algorithms or software that are central to the research but not yet described in published literature, software must be made available to editors and reviewers. We strongly encourage code deposition in a community repository (e.g. GitHub). See the Nature Portfolio guidelines for submitting code & software for further information.

Data

Policy information about availability of data

All manuscripts must include a data availability statement. This statement should provide the following information, where applicable:

- Accession codes, unique identifiers, or web links for publicly available datasets
- A description of any restrictions on data availability
- For clinical datasets or third party data, please ensure that the statement adheres to our policy

Because of participant confidentiality and privacy concerns, data are available upon written request. According to standard controlled access procedure, applications to use NHS/NHSII/HPFS resources will be reviewed by our External Collaborators Committee for scientific aims, evaluation of the fit of the data for the

proposed methodology, and verification that the proposed use meets the guidelines of the Ethics and Governance Framework and the consent that was provided by the participants. Investigators wishing to use NHS/NHSII/HPFS data are asked to submit a brief description of the proposed project (contact: nhsaccess@channing.harvard.edu). Investigators can expect initial responses within 4 weeks of request submission. Details are available on https://www.nurseshealthstudy.org/researchers and https://sites.sph.harvard.edu/hpfs/for-collaborators/. Source data supporting all our findings (Figures 2–3 and Supplementary Figures 1–3 and 5–6) are provided with this publication as a Source Data file.

Human research participants

Policy information about studies involving human research participants and Sex and Gender in Research.

Reporting on sex and gender	Both sexes (determined based on self-reporting) are included in this manuscript. Detailed information are shown in Table 1. We included sex as a covariate in our main analyses. In addition, we also conducted subgroup analysis by sex. The results were consistent in both groups.
Population characteristics	Participants in the discovery dataset were from the US, and participants in the replication dataset are from Spain. Details on the characteristics of the participants included in this manuscript are provided in Table 1.
Recruitment	Our samples are part of prospective matched case-control studies nested within the Nurses Health Studies' (NHS), NHSII and Health Professional Follow-up Study (HPFS). NHS, NHSII and HPFS are closed cohorts. NHS and NHSII include registered female nurses while HPFS includes male health professionals (veterinarians, dentists, pharmacists, optometrists, osteopath physicians, and podiatrists). Although the participants had a slightly higher socioeconomic status than did the general population and were mostly White, which may have initially affected generalizability, the population selection enhances internal validity because the health knowledge and commitment to research of the health professionals contributes to high-quality and complete self-reported health data as well as high follow-up rates. Although our participants are not a random U.S. sample, it seems unlikely that the biological relations in these participants will differ from the general population. The nurses and health professionals represent all but the poorest strata of society, and except for being predominantly Caucasian (reflecting registered nurses and health professionals at enrollment), have similar age and region- adjusted chronic disease rates to national rates, suggesting that our study is reasonably representative.
Ethics oversight	The study protocol for each cohort was approved by the institutional review boards (IRBs) of the Brigham and Women's Hospital, Harvard T.H. Chan School of Public Health, and participating registries. Completion of self-administered questionnaires and returns of blood samples were considered as implied consents by the IRBs.<br><br>The IRB of Hospital Clinic (Barcelona, Spain) approved the study protocol, and all participants provided written informed consent.

Note that full information on the approval of the study protocol must also be provided in the manuscript.

Field-specific reporting

Please select the one below that is the best fit for your research. If you are not sure, read the appropriate sections before making your selection.

☒ Life sciences ☐ Behavioural & social sciences ☐ Ecological, evolutionary & environmental sciences

For a reference copy of the document with all sections, see nature.com/documents/nr-reporting-summary-flat.pdf

Life sciences study design

All studies must disclose on these points even when the disclosure is negative.

Sample size	The sample size of the study was based on the number of participants that had available metabolomics data.
Data exclusions	For the analysis of mortality, we excluded participants reporting a history of cardiovascular disease (CVD) and cancer at blood collection or lost to follow-up after blood draw. For the analysis of longevity, we excluded participants who were alive but did not achieve longevity (living to age 85 years) at the end of follow-up.
Replication	The results for the associations of metabolites and multi-metabolite profile score with mortality and longevity in NHS/NHSII/HPFS /were independently replicated in an independent dataset, the PREDIMED study.
Randomization	Not applicable. This study is an observational study. There are no experimental groups or interventions in the study.
Blinding	Not applicable because this is an observational study without any interventions. Physicians who reviewed the medical records were unaware of the exposure information.

Reporting for specific materials, systems and methods

We require information from authors about some types of materials, experimental systems and methods used in many studies. Here, indicate whether each material, system or method listed is relevant to your study. If you are not sure if a list item applies to your research, read the appropriate section before selecting a response.

Materials & experimental systems

n/a	Involved in the study
☒	☐ Antibodies
☒	☐ Eukaryotic cell lines
☒	☐ Palaeontology and archaeology
☒	☐ Animals and other organisms
☒	☐ Clinical data
☒	☐ Dual use research of concern

Methods

n/a	Involved in the study
☒	☐ ChIP-seq
☒	☐ Flow cytometry
☒	☐ MRI-based neuroimaging
